# Left-Handed Helices in DNA Nanotechnology

**DOI:** 10.1021/jacsau.6c00027

**Published:** 2026-02-23

**Authors:** Sangeetha Selvam, Johnsi Mathivanan, Arun Richard Chandrasekaran

**Affiliations:** † The RNA Institute, 1084 University at Albany, State University of New York, Albany, New York 12222, United States; ‡ Department of Nanoscale Science and Engineering, University at Albany, State University of New York, Albany, New York 12222, United States

**Keywords:** DNA nanotechnology, DNA double helix, left-handed DNA, DNA topology, Z-DNA, L-DNA, DNA origami

## Abstract

DNA nanotechnology involves the use of DNA as a material to build nanoscale shapes and structures. This strategy typically uses the conventional right-handed B-form DNA for nanoscale construction. In some cases, alternate nucleic acid structures, such as those that form left-handed helices, are used in the design and assembly of nanostructures. Creation of left-handed helices for nanostructures uses Z-DNA, a left-handed structure, or L-DNA strands that result in the formation of a left-handed duplex. In addition, structures that have a global left-handed helicity but underlying right-handed B-DNA units have also been created. This perspective discusses the left-hand helices used in DNA nanotechnology, their applications, and their future impact.

## Graphical abstract

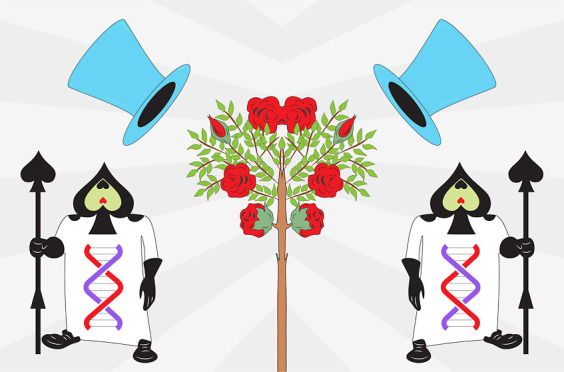

## Introduction

Construction using DNA to create nanoscale structures relies on the precise design and helical parameters of double-stranded DNA.^1,2^ In most DNA nanostructures, the underlying DNA is the typical right-handed B-DNA.^3^ While the right-handed helicity is the predominant structure, there are examples of nanostructures constructed using left-handed DNA helices (Figure 1). DNA structures can adopt a left-handed structure when containing specific sequences and under certain environmental conditions.^4^ For example, stretches of GC sequences can lead to the formation of Z-DNA, a left-handed zigzag double helical structure of DNA.^5^ Further, Z-DNA is also promoted by counterions such as cobalt hexammine.^6^ In addition to naturally occurring left-handed helices, the development of synthetic nucleic acid analogs has allowed the creation of a variety of DNA structures containing left-handed helical domains. L-DNA, which contains left-handed deoxyribonucleotides, can assemble into a left-handed DNA duplex, resulting in a mirror image structure of a typical B-DNA right-handed structure.^7^ Since L-DNA is the mirror image of conventional D-DNA (the typical right-handed DNA), it resists nuclease digestion and nonspecific binding to natural proteins, and it cannot hybridize with any D-nucleic acids.^8,9^ These biocompatible nucleic acid nanostructures maintain similar physical and chemical properties as D-DNA while exhibiting opposite chirality.^10^

**Figure 1:**
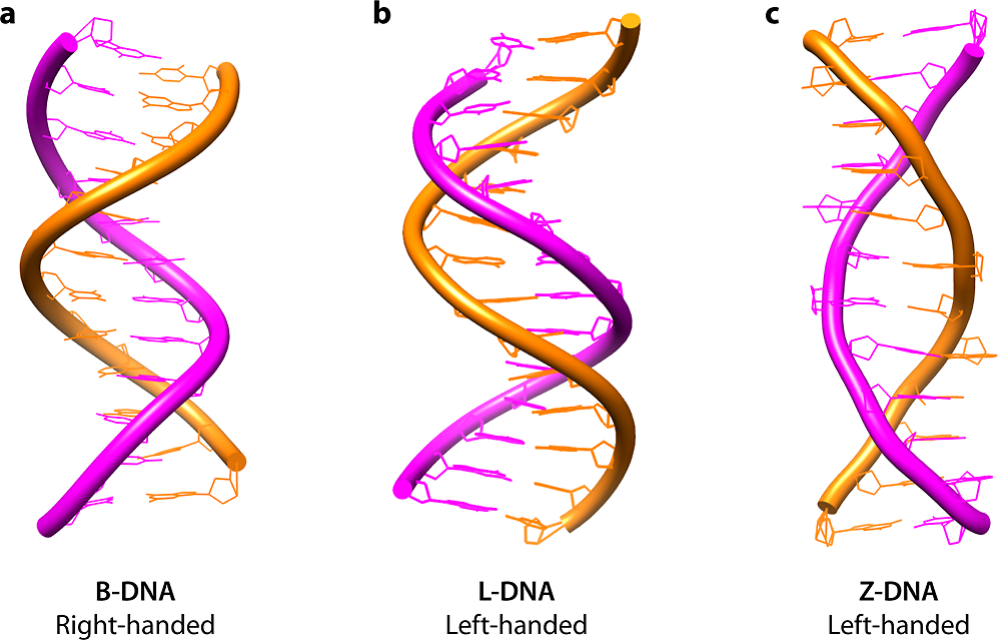
Handedness in DNA helices. (a) A typical B-DNA right-handed duplex (also referred to as D-DNA). PDB: 1BNA. (b) A left-handed duplex formed from L-DNA strands. PDB: 6L97. (c) A left-handed Z-DNA duplex. PDB: 4OCB.

Left-handed DNA structures from both naturally occurring DNA and artificial analogs have been used in the construction of DNA nanostructures. In these structures, the left-handedness of the helices provides additional characteristics and functionalities to the structure, thus expanding their applications. In addition, DNA nanostructures can be constructed with strand routing patterns that create structures with a global left-handed twist. While the use of left-handed DNA is well studied in mirror-image i-motifs^11^ and G-quadruplexes,^12^ their effect on the structure and function of nucleic acid nanostructures is only now beginning to be explored. Such left-handed helical DNA nanostructures have found applications in biosensing, molecular computation, and drug delivery.^13^ In this perspective, we discuss the assembly and functionality of DNA nanostructures containing left-handed DNA helices as well as structures that are globally left-handed. We define the scope of this perspective to include only those structures that contain left-handed helices. Different chiral arrangements created by DNA scaffolds^14,15^ and chiral molecules developed from DNA^16,17^ are reviewed elsewhere.^18,19^

## Static Structures Involving Left-Handed DNA

The simplest route to creating nanostructures with left-handed helices is to replace a typical D-DNA component strand with an L-DNA strand. One of the earliest examples of an L-DNA-based nanostructure is a four-arm junction (Figure 2a).^20^ L-DNA junctions showed a similar melting temperature (∼45 °C) to that of D-DNA junctions. However, the L-DNA junction was resistant to exonuclease, while the D-DNA junction was completely digested under the same conditions. DNA nanotubes^21^ and 2D crystals^22^ assembled from a single DNA strand were also assembled using an L-DNA strand. Nanotubes formed from L-DNA strands exhibited a right-handed chirality, while the D-DNA nanotubes were left-handed, indicating that the overall chirality of the nanotubes is related to the intrinsic chirality of the DNA helix (Figure 2b). On incubation with a DNA exonuclease, the L-DNA nanotubes remained intact after enzymatic digestion, while the D-DNA nanotubes were degraded. Further, palindromic segments within the single strand allow it to self-hybridize and form a pseudocontinuous duplex that further self-assembles into 2D arrays by sticky-end cohesion, with rhombus-shaped cavities.

**Figure 2:**
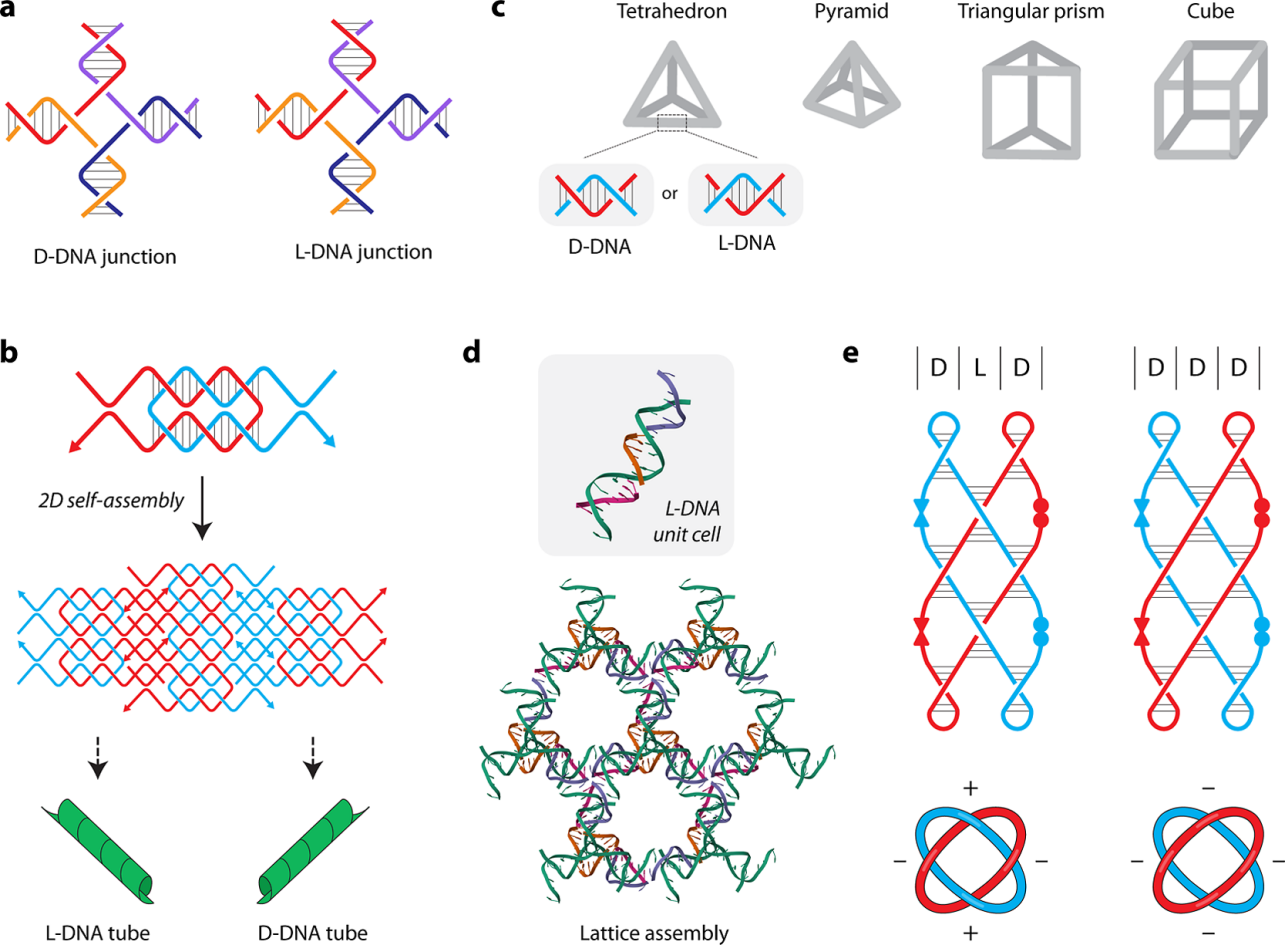
Static structures involving left-handed DNA. (a) Right- and left-handed 4-arm DNA junctions.^20^ (b) Nanotubes formed from D-DNA and L-DNA strands.^20^ (c) DNA polyhedra built using L-DNA strands. The gray lines depict double-stranded edges that are either right- or left-handed when assembled from D-DNA or L-DNA strands, respectively.^23^ (d) A self-assembled crystal formed from the L-DNA motif.^26^ (e) Weaved structures with positive or negative nodes assembled from L-DNA and D-DNA chimera strands.^30^

Other nanostructures such as polyhedra and cubes have also been created by using L-DNA (Figure 2c). For example, a DNA cube was constructed from six DNA strands that contained either D-DNA or L-DNA components, resulting in dimensions of 10 × 21 × 21 base pairs in edges.^23^ When tested in 10% serum, the L-DNA cube was almost fully intact for more than 12 h, while the D-DNA cube was completely degraded within 10 min. In a similar strategy, DNA tetrahedra constructed from L-DNA showed enhanced serum stability. Specifically, L-DNA tetrahedra with 17 bp edges were more biostable compared to L-DNA tetrahedra with 30 bp edges, consistent with previous reports on size-dependent biostability of DNA tetrahedra.^24^ A library of DNA nanostructures including a pyramid, triangular prism, cube, and rugby-ball-like construct was assembled from four different DNA backbones including D-DNA, L-DNA, 2′-OMe-RNA, and 2′-F-RNA (2′-deoxy-2′-fluoro-nucleosides).^25^ The nucleic acid structures with modified sugar backbones showed gel mobility and hydrodynamic sizes similar to those of D-DNA nanostructures, suggesting that replacing the typical DNA strand with modified components results in proper assembly of the nanostructures.

The use of L-DNA sequences has been extended to 3D DNA crystals self-assembled from DNA motifs.^26^ The motif contains four double helical domains connected by a single strand that weaves through the four domains, forming 6 base pairs within each domain. The motifs are tailed by sticky ends that create a matrix of layers that are organized and dictated by a series of junctions. Specifically, the array contains cavities that are roughly 4 × 2 × 5 nm in size. These crystals were also assembled using L-DNA strands, resulting in mirror image crystals with sequences identical to that of the D-DNA crystal but are resistant to nucleases (Figure 2d). When incubated with DNase I enzyme at 37 °C, the D-DNA crystals degraded completely within 6 h, while the L-DNA crystals showed no apparent change in morphology throughout the entire time course, indicating their complete resistance to DNase I digestion.

Assembly of topological constructs involves the creation of nodes that typically correspond to a half-turn of DNA (∼6 bp), resulting in crossing points in a knot or a catenane. One approach to achieving this involves the use of left-handed Z-DNA strands by controlling the geometry and placements of junctions and knots.^27–29^ However, the use of Z-DNA has some limitations including the lack of sequence diversity for Z-DNA formation, the difficulty of forming Z-DNA within very short segments, and the difference in structure of Z-DNA not being an exact mirror image of the right-handed B-DNA. These limitations can be overcome by the use of mixed chirality of nucleotides within B-DNA backbones, allowing control over the global handedness.^30^ Strategic placement of D-nucleotides and L-nucleotides at the nodes of a B-DNA structure allowed the construction of a planar woven arrangement with specific nodes allowed by left-handed domains (Figure 2e). Controlling the node handedness is highly crucial for the formation of left-handed topologies and offers a versatile approach compared to the use of noncanonical nucleic acid structures. On adjusting the junction nodes and variation of the base pair length, various knots and catenanes with desired topology can be achieved with alternating positive and negative nodes.

Other non-natural nucleic acid polymers also provide the flexibility to build right- and left-handed geometries. Glycerol nucleic acid (GNA) offers unique structural and chemical properties, including the possibility to construct right- and left-handed helices.^31^ Custom-synthesized (*R*)- and (*S*)-GNA can self-assemble into immobile 4-arm junctions similar to DNA but with enhanced thermal stability.^32^ On comparing the melting transition between GNA-based junctions with the DNA version, GNA structures displayed a significantly higher melting temperature (76 °C) than their DNA counterpart (37 °C), indicating superior thermal stability. The structural stability and the flexible chirality of GNA make it a promising candidate for DNA nanotechnology and molecular scaffolding and for future nanotechnological applications.

## Dynamic Devices Involving Left-Handed Helices

Z-DNA sequences can play a crucial role in gene regulation and expression,^33–35^ and the occurrence of B–Z transitions can be induced by external factors such as high salt concentrations,^36^ negative supercoiling,^37^ and changes in specific metal ion concentrations.^38^ At the molecular level, the transition alters the handedness of the nucleic acid helix and exposes unique structural features for the building of nanoscale architectures. A nanomechanical device assembled from two double crossover (DX) molecules was operated by interconverting a double-stranded domain to be right- or left-handed (Figure 3a).^39^ The two DX molecules were connected by a 20 bp double-stranded region that contains a proto-Z sequence d­(CG)_10_ that can be converted to left-handed Z-DNA at high ionic strength. Further, the 5-position of cytosine was methylated in the proto-Z sequence since the modification is known to increase the propensity of DNA to undergo the B–Z transition.^40^ In the B-form, the connecting segment is right-handed and exists under low-salt conditions. Transition to the left-handed Z-form was achieved by the addition of hexamminecobalt­(III) chloride, leading to the rotation of the device by ∼128° along the axis of the double-stranded connector. This framework for the relative positioning of short helical B–Z transition components could be adapted into other nanostructures for directed motion.

**Figure 3:**
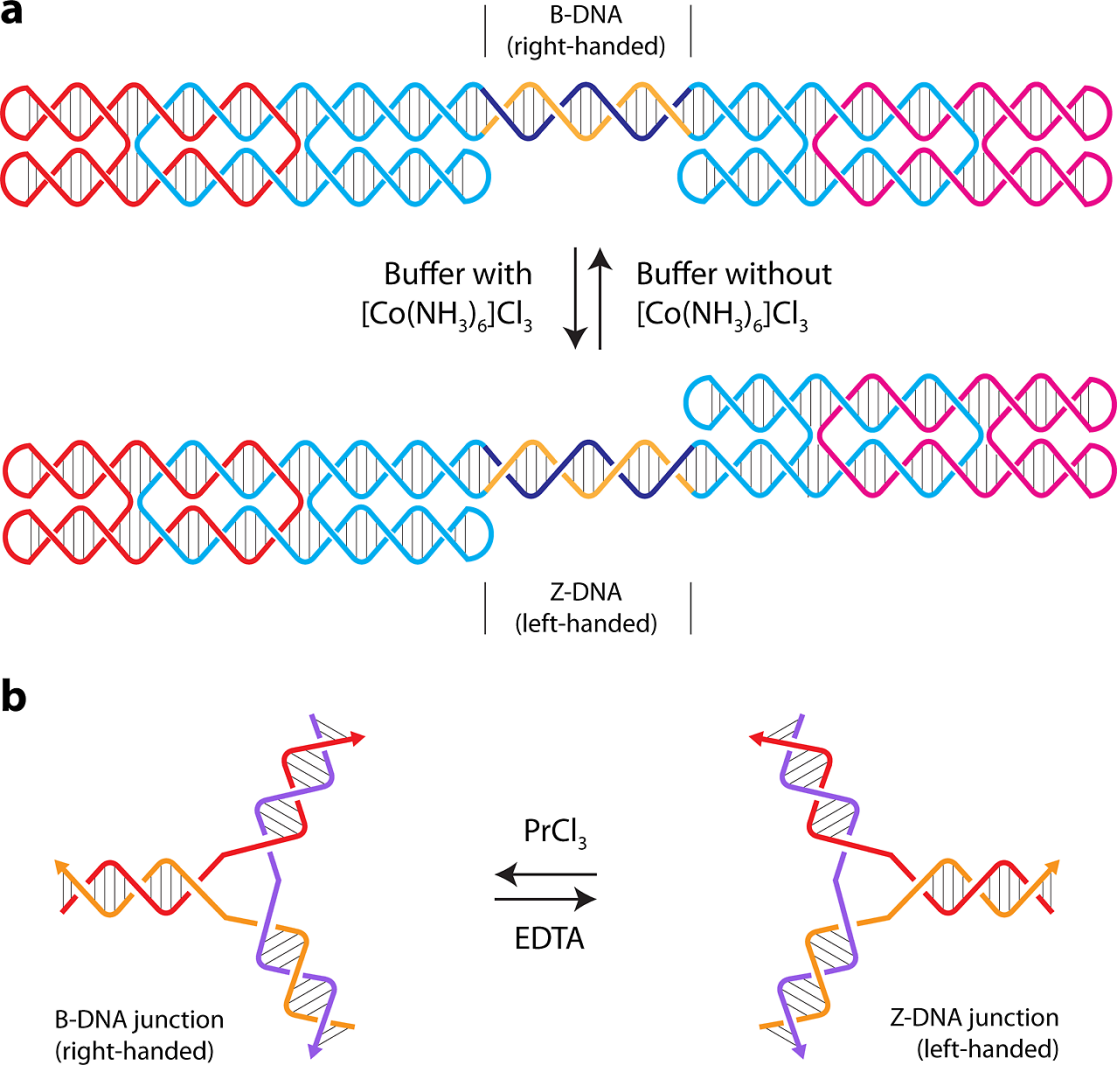
Dynamic devices involving left-handed helices. (a) A B–Z DNA device interconverts between right- and left-handed helices on the addition of cobalt hexammine.^39^ (b) A 3-arm junction changes from right-handed B-DNA to left-handed Z-DNA in the presence of praseodymium chloride.^43^

B–Z transition in 3-arm DNA junctions has also been induced by lanthanide complexes such as lanthanum (La^3+^),^41^ cerium (Ce^3+^),^42^ and praseodymium (Pr^3+^) (Figure 3b).^43^ The higher electropositive character and significant hydration sphere of lanthanide series salts provide stability to the inverted phosphate groups in Z-DNA, thus promoting the B–Z transition.^44^ The concentration of the counterion and the number of thymines in the loop of the 3-arm junction influenced this character. A 3 T loop was more sensitive to the B–Z transition compared to a 5 T loop. Further, while 5 mM LaCl_3_ and 7.5 mM CeCl_3_ were enough to induce a left-handed Z-conformation, 10 mM of PrCl_3_ was required for a stable Z-DNA conformation of the 3-arm junction. The critical aspect of these nanostructures is that the handedness can be reversed by using chelating agents such as EDTA for restoring the canonical B-DNA conformation.

In addition to the interconversion between right- and left-handed helices, the chiral differences of component DNA strands can also be used in controlling DNA circuits. Introduction of short L-DNA sequences to the ends of D-DNA strands enhanced nuclease resistance of DNA circuits in biological fluids.^45^ These heterochiral nucleic acid structures with left-handed helical segments shield the D-DNA segments from exonuclease degradation, improving the stability of DNA-based nanostructures. This L-DNA-based “toecap” system was used to demonstrate a heterochiral translator system in serum-supplemented media.^46^

## Topologically Left-Handed DNA Nanostructures

The construction of left-handed DNA nanostructures can be achieved through natural and synthetic design principles. To avoid the use of Z-DNA in topological constructs, multiple right-handed B-DNA half-turn domains were combined to result in a structure called switchback DNA.^47,48^ In switchback DNA, a series of B-DNA half-turns are aligned laterally, where the helical axis of the half-turn domains is perpendicular to the axis of the full structure.^48,49^ The switching back of the backbone after each half-turn results in a structure that is a globally left-handed helix with the two strands arranged parallel to each other, but the underlying half-turns are typical right-handed B-DNA with antiparallel strand orientations (Figure 4a). Similar to switchback DNA, switchback RNA molecules can also be constructed with a global left-handed helicity.^50^ Switchback DNA is more nuclease-resistant compared to duplex DNA against enzymes such as DNase I and T5 exonuclease and elicits minimal immune response compared to duplex DNA. Further, based on the sequence arrangement of switchback DNA, the structure is hypothesized to be a potential alternate structure in short tandem repeats in the human genome.

**Figure 4:**
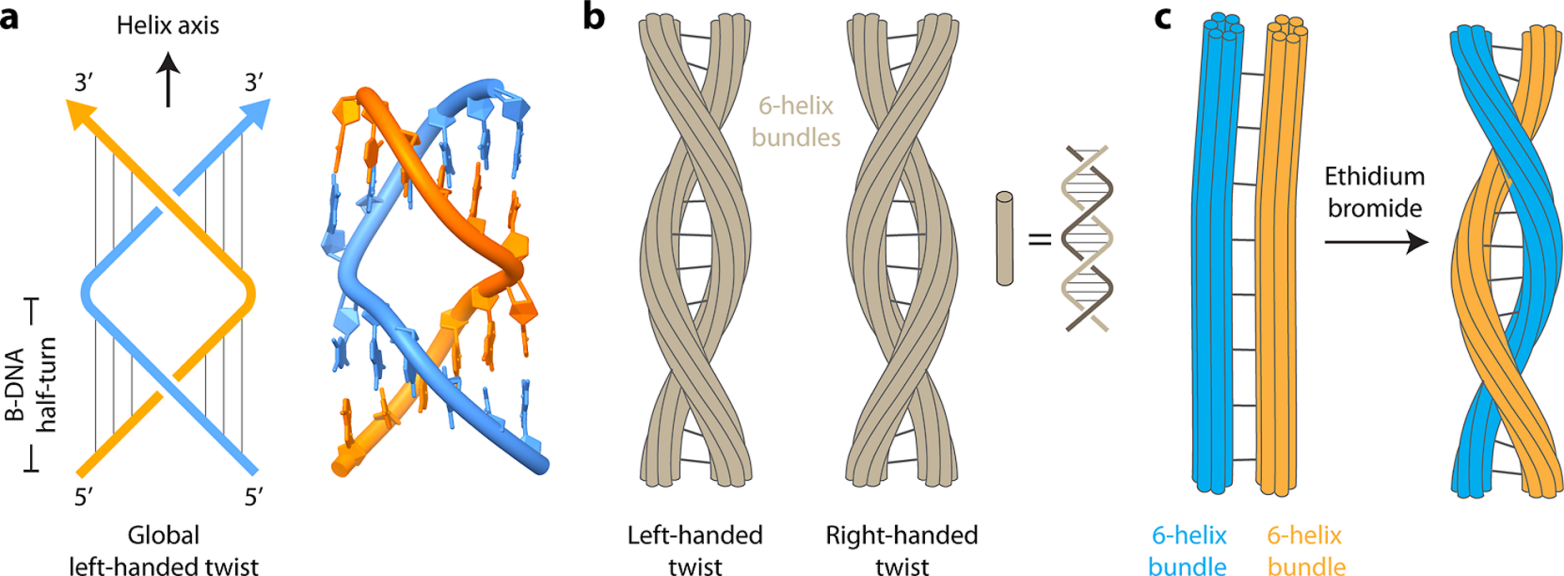
Topologically left-handed DNA nanostructures. (a) Switchback DNA structure with a global left-handed twist and parallel strand orientation. The underlying half-turn domains are typical right-handed B-DNA.^49^ (b) *Meta*-DNA structures formed from 6-helix bundles that can be assembled to form large-scale left- or right-handed meta-helices. The individual duplexes in the bundles are typical right-handed B-DNA.^51^ (c) Interconnected 6-helix bundles in a DNA nanorail undergo a twist to form left-handed helices on the addition of intercalators such as ethidium bromide.^52^

Larger topologically left-handed structures can be created by specific designs of DNA origami bundles, termed *meta*-DNA.^51^ In *meta*-DNA, 6-helix bundles act as modular building blocks, mimicking single-stranded DNA behavior. Two 6-helix bundle units can hybridize by *meta*-base pairs with controlled handedness and helical pitches, thus creating 1D structures that resemble a left-handed meta-helix (Figure 4b). Such *meta*-DNA structures can extend to micrometer-scale architectures and metamaterials.

A global left-handed helical twist can also be induced by DNA intercalators such as ethidium bromide and SYBR Green I.^52^ The effect of global twist was studied in a DNA nanorail structure assembled from two 6-helix bundles connected by multiple crossovers. Lengthening of the DNA on addition of intercalators was accommodated by unwinding of the bundles, resulting in a left-handed twist of the nanorails (Figure 4c). Left-handed global twisting of DNA nanorails increased as a function of the intercalator concentration, with saturation at higher intercalator concentrations. Molecular dynamic simulations of DNA origami structures indicate changes in DNA base pair geometry due to intercalation.^53^ In the case of DNA origami ring structures, binding of ethidium bromide to DNA induced negative torsion, resulting in the positive supercoiling of the structures. Understanding such chirality assessment would be useful to refine nanostructure designs.

## Applications

The stability and biocompatibility of left-handed helices make them highly suitable for applications in drug delivery, biosensing, and molecular computing.^54,55^ The major advantage of using left-handed helices in nucleic acid nanostructures is their resistance to nuclease degradation combined with their higher melting temperatures. L-DNA nanostructures can enhance drug delivery efficiency by enabling the targeted transport of drug molecules while maintaining their structural integrity in biological environments. Further, these structures showed enhanced cellular uptake without needing transfection agents, ensuring prolonged intracellular functionality.^23,56,57^ L-DNA nanostructures have been used to deliver nucleic acid cargos such as DNAzymes,^23^ aptamers,^57^ siRNAs,^58^ and antisense oligonucleotides (ASOs) (Figure 5a).^59^ Delivery of DNAzyme 8–17 using an L-DNA nanocube resulted in highly efficient silencing of miR-21 in MCF-7 cancer cells.^23^ Similarly, L-DNA tetrahedra delivered the AS1411 aptamer with greater efficacy than D-DNA carriers, improving cancer cell targeting and cytotoxicity while reducing off-target effects.^57^ L-DNA tetrahedra have also been used as a platform for kidney-targeted cytosolic delivery of p53 siRNA to treat acute kidney injury (AKI) in mice.^58^ Only the mirror-image DNA tetrahedron accumulated in the kidney after intravenous injection due to its low serum protein opsonization. Due to their smaller size, these structures are compatible with glomerular filtration and are efficiently taken up by renal tubular cells. Further, gene silencing was achieved at a much lower dose for siRNA delivered by L-DNA tetrahedra compared to naked siRNA with reduced apoptosis in kidney tissue. L-DNA cubes containing ligands that target endocytic receptors in brain microvascular endothelial cells have been used to cross the blood–brain barrier in a protein-corona-assisted manner.^59^ When loaded with ASO targeting a glioblastoma gene, the DNA cube effectively delivered it into mouse brain tumor tissue, resulting in gene downregulation and antitumor effects.

**Figure 5:**
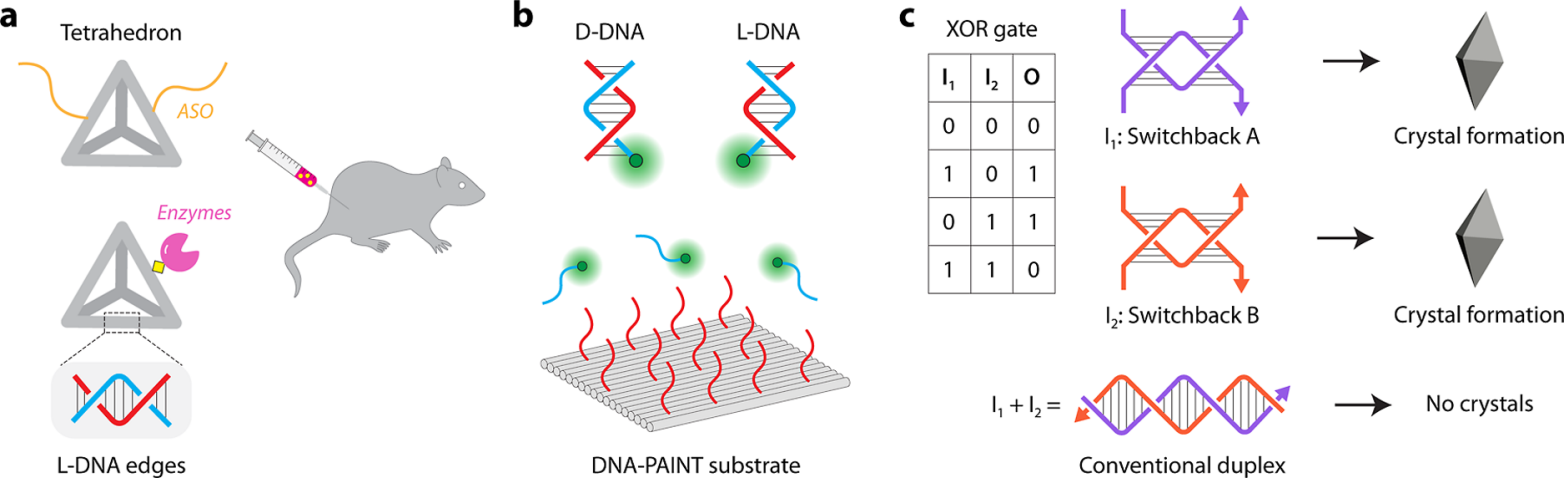
Applications of left-handed DNA nanostructures. (a) DNA polyhedra constructed from L-DNA strands are used in drug delivery. (b) DNA origami structures containing L-DNA probes are used in the DNA PAINT strategy for biosensing. (c) Strand displacement between switchback DNA and conventional duplex is used in logic-gated engineering of DNA crystals.

L-DNA polyhedra have also been used to deliver small molecules and proteins. L-DNA tetrahedra efficiently delivered doxorubicin to tumor sites with improved cellular uptake comparable to PEGylated liposomes, and L-DNA tetrahedra injected into mice remained in the bloodstream even after 6 h.^56^ A streptavidin-functionalized L-DNA tetrahedron was used to deliver the enzymes caspase-3, Cre recombinase, and β-galactosidase into cells without any loss in activity (Figure 5a).^25,60^ In mice, the streptavidin-functionalized L-DNA tetrahedra selectively accumulated in tumors and enabled tumor-specific enzyme delivery, leading to targeted tumor growth suppression without any adverse effect on healthy cells. Comparison of different backbone modifications in tumor-bearing mice indicated that L-DNA (specifically pyramid-shaped structures) exhibited the highest tumor specificity when compared to D-DNA, 2′-OMe-RNA, and 2′-F-RNA structures.^25^ With the serum stability offered by mirror-image DNA, these L-DNA structures provide efficient cancer cell uptake and macrophage evasion.

In biosensing applications an L-DNA cube was used to encapsulate the D-DNA probes for DNase-activated microRNA detection with high signal-to-noise ratios.^23^ In addition, L-DNA oligomers have been used in DNA-PAINT and show the same specificity and multiplexing capability as D-DNA-PAINT, but with improved visualization of target molecules and substantially reduced background signal.^61^ This strategy has been incorporated into DNA origami PAINT for rapid and efficient imaging of neuronal interactomes with 15 nm spatial resolution (Figure 5b).^62^

The use of topologically left-handed helices such as in switchback DNA allows affinity-based strand displacement reactions in contrast to toehold-based strand displacement. Addition of a duplex complement to a switchback DNA can displace the switchback complement, resulting in a conventional duplex.^63^ This strategy has been used in controlling strand displacement reactions in the operation of a DNA tweezer.^64^ Further, this difference in affinity between switchback DNA and conventional DNA also allows the operation of logic gates in 3D crystals self-assembled by sticky end cohesion of switchback DNA starting units (Figure 5c).^65,66^ Homodimer switchback DNA tailed with sticky ends resulted in hierarchical assembly of the motifs, leading to crystal formation. However, if sequences in two switchback DNA motifs were complementary, then the component strands of the switchback DNA motifs would form a conventional duplex, preventing crystal formation.

## Outlook

The use of left-handed helices in DNA nanotechnology aids in the development of enhanced structures, as well as nanoscale scaffolds with unusual geometries and linkages. Understanding the effect of left-handed helices on the assembly and biological applications requires a further systematic analysis. For example, the chirality of DNA has been shown to have an influence on the behavior of oligonucleotides inside cells. Single-stranded G-rich L-RNAs are more cytotoxic than their D-DNA/RNA counterparts,^67^ but this effect has not been shown for L-DNA structures. Given that Z-DNA is known to play biological roles, any adverse effects of the use of left-handed DNA seems minimal.^68^ In addition, protein corona formation is a key factor in the biomedical applications of DNA nanostructures. The chemical identity of the nucleic acid used in constructing the nanostructure has been shown to influence the type of protein corona formation, causing a higher accumulation of L-DNA tetrahedra in the kidney, while 2′-F-RNA tetrahedra were found in the liver.^58^ A comparison of nucleotide analogs in different cell types would be useful to rationally choose the modifications required for the construction of a drug carrier for specific cells or tissues.

Developing switchable conformations of nucleic acid nanodevices is of critical importance for biosensing and diagnostic applications. The B–Z transition represents a molecular mechanism for integrating dynamic conformational switching into DNA-based nanostructures,^39^ where chirality can be easily modulated using ionic conditions or metal-ion binding at the nanoscale level. Reversible B–Z reconfiguration of branched junctions using rare earth elements and EDTA has been shown^43^ but has not been demonstrated in larger nanostructures. Applications of L-DNA can be combined with the programmability of DNA nanostructures in the future, such as the chirality-specific sorting of carbon nanotubes,^69^ and in data storage, where the identity of chimeric D-DNA/L-DNA inputs can be used to encode different information or messages depending on the chirality of reading.^70^

There are several developments in nucleic acid chemistry and oligonucleotide applications that can be incorporated into DNA nanostructures. For example, L-DNAzymes that are nuclease resistant can be used in metal ion sensing.^71^ Further, other nucleotide modifications such as 2′-*O*-methyl can be combined with L-DNA and L-RNA to make the nucleic acid structures with enhanced thermal stability.^72^ 2′-F-araC and 2′-F-riboG modifications that induce the formation of Z-DNA under low ionic strength in duplex DNA could be incorporated into DNA nanostructures for reconfiguration under mild ionic conditions.^73^ In addition, the use of specific DNA and RNA sequences allows temperature-based control over helix handedness by B–Z or A–Z transition for nucleic acid devices.^74^ DNA machines and reconfigurable devices can also be operated using DNA polymerases that can act on L-DNA duplexes^75^ and DNA ligase made of d-amino acids that can act on L-DNA.^76^ In addition, mirror-image aptamers made from chirally inverted nucleic acids are nuclease-resistant and exceptionally biostable, opening up opportunities for unique applications. One important factor in the use of L-DNA in nanotechnology is the cost involved in the synthesis of L-DNA strands. While currently more expensive than conventional DNA, several recent developments in the synthesis and characterization of left-handed DNA could potentially make the use of L-DNA more accessible. For example, directed evolution and selection of biostable L-DNA aptamers can be achieved with a mirror-image DNA polymerase,^77^ and enzyme-free primer extension of PNA can be used to sequence D-DNA or L-DNA.^78,79^ Further, reusing or recycling the same set of strands in the construction of nanostructures^80,81^ could reduce the overall cost involved. Microarray-based synthesis of L-DNA^82^ might also be an alternative to synthesizing L-DNA strands for nanotechnology applications.

Overall, this perspective highlights the versatility of DNA as a highly programmable scaffold. The feasibility of constructing synthetic topological constructs with different handednesses adds to the programmability of nucleic acid nanostructures for various applications. The advances discussed here provide a toolkit for designing synthetic topological architectures and nucleic acid-based functional materials.
